# Hedgehog morphogen gradient is robust towards variations in tissue morphology in *Drosophila*

**DOI:** 10.1038/s41598-023-34632-8

**Published:** 2023-05-25

**Authors:** Giulia Pierini, Christian Dahmann

**Affiliations:** 1 School of Science, Technische Universität Dresden, 01062 Dresden, Germany; 2 Cluster of Excellence Physics of Life, Technische Universität Dresden, 01062 Dresden, Germany

**Keywords:** Cell biology, Developmental biology

## Abstract

During tissue development, gradients of secreted signaling molecules known as morphogens provide cells with positional information. The mechanisms underlying morphogen spreading have been widely studied, however, it remains largely unexplored whether the shape of morphogen gradients is influenced by tissue morphology. Here, we developed an analysis pipeline to quantify the distribution of proteins within a curved tissue. We applied it to the Hedgehog morphogen gradient in the *Drosophila* wing and eye-antennal imaginal discs, which are flat and curved tissues, respectively. Despite a different expression profile, the slope of the Hedgehog gradient was comparable between the two tissues. Moreover, inducing ectopic folds in wing imaginal discs did not affect the slope of the Hedgehog gradient. Suppressing curvature in the eye-antennal imaginal disc also did not alter the Hedgehog gradient slope but led to ectopic Hedgehog expression. In conclusion, through the development of an analysis pipeline that allows quantifying protein distribution in curved tissues, we show that the Hedgehog gradient is robust towards variations in tissue morphology.

## Introduction

A fundamental aspect of animal development is the timely provision of positional information to cells within tissues. Positional information can be provided by the local expression of secreted signaling molecules (morphogens)^1^, which then disperse throughout the receiving tissue through diverse mechanisms, including diffusion, dispersal on vesicles, transcytosis and cellular protrusions^2–7^. Morphogen dispersal and degradation lead to the formation of a concentration gradient in the receiving tissue. Cells in the receiving tissue read out the concentration gradient through specific receptors and respond by activating or suppressing the expression of target genes in a threshold-like manner, thereby contributing to determining the fate of cells^8–12^. Cell fate may entail the specification of mechanical properties, which can lead to a change in the shape of cells. Coordinated cell shape changes in turn drive the three-dimensional deformation of the tissue^13–15^. A common three-dimensional deformation is epithelial folding, which, for example, is important for embryonic gastrulation, neural tube formation and gut development^14,16–19^.

Such alterations of the epithelial morphology create a different environment for morphogen gradient formation. Whether and how patterning through morphogen gradients is robust towards fluctuations in biological conditions is a fundamental question in the field^20–22^. Theoretical and experimental studies have shown that patterning through morphogen gradients is robust: morphogen gradients often scale with tissue size^22–26^, with relevant exceptions^27^, and their formation is not affected by different levels of morphogen production^28,29^ or by a source expanding in time^30^. Recently, it has been proposed that cell morphology plays a fundamental role in achieving patterning precision^31^. However, it remains largely unexplored whether changes in tissue morphology, such as epithelial folding, can impact on the robustness of the patterning. For instance, tissue shape could affect the distribution of the morphogen by affecting its free diffusion, locally concentrating the morphogen^32^, changing the distribution of the receptors or altering the effective tissue size.

**Figure 1:**
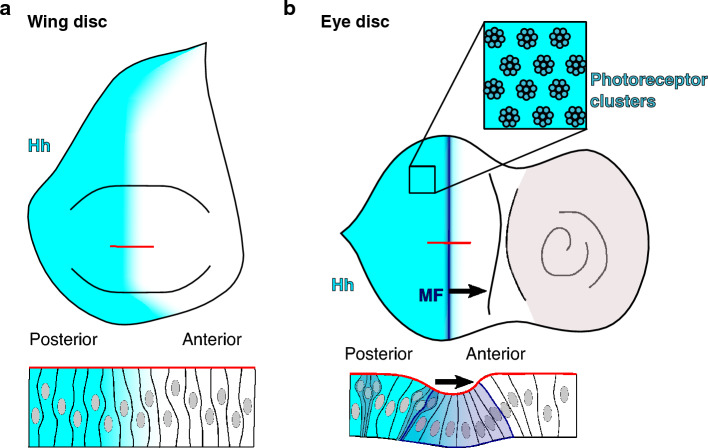
Schematic representation of the tissue morphology and the Hedgehog gradient in the wing and eye discs in *Drosophila.* (**a**) Wing disc where the Hedgehog gradient is shown in cyan. Top view (top) and side view (bottom) are shown. (**b**) Eye disc where the Hedgehog gradient (cyan), the morphogenetic furrow (blue) and the photoreceptor clusters (dark cyan) are shown. Top view (top), side view (bottom). Black arrows indicate the direction of the movement of the morphogenetic furrow over time (from posterior to anterior).

Common model systems to study tissue patterning and morphogen gradients are larval *Drosophila* imaginal discs. Imaginal discs are epithelial monolayers that give rise to adult body parts such as the wing, eye, antenna and leg. Growth and patterning of larval imaginal discs are largely regulated by gradients of morphogens, including Decapentaplegic, Wingless and Hedgehog (Hh)^33–40^. In the wing imaginal disc (henceforth wing disc), Hedgehog is expressed only in cells of the posterior compartment. It is secreted by posterior cells and disperses to adjacent anterior cells, where it forms a concentration gradient (Fig. 1a)^34,40–43^. Different dispersal mechanisms of Hedgehog have been proposed, including glypican-mediated diffusion^44^, transport on extracellular vesicles^45^ and cell–cell contact via basal cytonemes^46,47^. The Hedgehog concentration gradient results in a threshold-dependent induction of target gene expression^48^. Likewise, in the part of the eye-antennal imaginal disc that gives rise to the eye (henceforth eye disc), Hedgehog is expressed in posterior cells (Fig. 1b)^49^. During eye disc development, a differentiation wave sweeps from posterior to anterior, transforming the initial epithelial cells into photoreceptors and associated cells^50,51^. The movement of the differentiation wave is driven by a positive feedback loop, where the differentiating photoreceptor cells start to express Hedgehog^52^. Hedgehog moves anteriorly, leading to the differentiation of a new row of cells into photoreceptors. The differentiation wave is preceded by a transient fold, termed morphogenetic furrow (MF), whose formation and movement depend on Hedgehog signal transduction^53–55^. The morphogenetic furrow is positioned at the boundary between the Hedgehog source and the receiving tissue (Fig. 1b). Thus, the Hedgehog gradient forms across a fold. This contrasts the situation in the wing disc, where the Hedgehog gradient forms across a flat tissue (Fig. 1a).

Here, we sought to test the possible influence of tissue folding on the Hedgehog gradient by quantifying and comparing Hedgehog distribution in eye and wing discs. Quantification of protein abundance based on immunohistochemistry usually involves the arbitrary choice of an image plane for quantification or a projection of an image stack^56^. These methods, however, might lead to artifacts in the case of curved tissues. On one hand, in a curved tissue, a single x–y plane of the image corresponds to different apicobasal locations of the cells. On the other hand, performing simple projections over the z-axis leads to the inclusion of areas external to the tissue of interest. Therefore, we developed a standardized image analysis pipeline to enable the quantification of protein abundance in both flat and curved tissues. We then applied this image analysis pipeline to quantify and compare Hedgehog protein abundance in wing and eye discs. Moreover, we modified the tissue shape by introducing a fold in wing discs and by locally suppressing the morphogenetic furrow in eye discs, and analyzed the consequences on Hedgehog protein distribution. Our results show that, under our experimental conditions, the Hedgehog gradient shape is robust towards tissue folding. However, suppression of the morphogenetic furrow by altering the cells’ cytoskeleton results in the expansion of the Hedgehog source, indicating a possible link between the mechanical properties of cells and cell differentiation in the eye disc.

## Results

### Development of an analysis pipeline to quantify a morphogen gradient in curved epithelia

**Figure 2:**
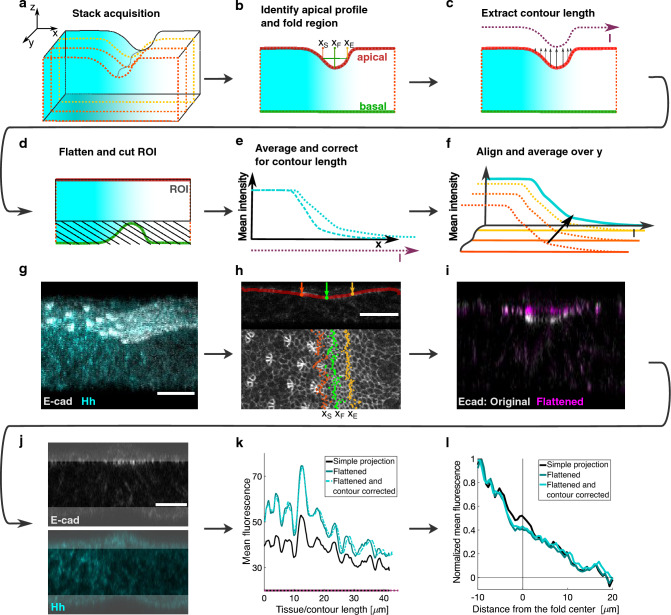
Pipeline for analyzing protein distribution in curved tissues. (**a-f**) Schematic representation of the analysis pipeline: (**a**) Three-dimensional image of a tissue showing a graded protein distribution in cyan. (**b–d**) Single x–z plane, showing the protein distribution, the apical and basal edge, and start (${\text{x}}_{{\text{s}}}$), center (${\text{x}}_{{\text{F}}}$) and end (${\text{x}}_{{\text{E}}}$) of the fold (**b**); extracted contour length (dotted line) and direction of image deformation (**c**); the flattened tissue and region excluded from quantification as black lines in (**d**) (ROI: region of interest). (**e**) Quantification of the protein distribution before and after correction for the contour length. (**f**) Alignment to the reference (fold location) and average over y. (**g–l**) Application example of the analysis pipeline. (**g**) 3D-reconstruction from a z-stack of images of an eye disc immunostained for E-cadherin and endogenous Hh. (**h**) Top: Identification of the apical edge and fold descriptors: start (red, ${\text{x}}_{{\text{s}}}$), center (green,${\text{x}}_{{\text{F}}}$) and end (yellow,${\text{x}}_{{\text{E}}}$) of the fold. Bottom: overlay of fold descriptors (as in the top) with a maximum z-projection of the E-cadherin signal. (**i**) Overlay of the E-cadherin signal of a single x–z plane before and after flattening (original and flattened, respectively). (**j**) Single x–z plane in the two channels (E-cad and Hh) from the z-stack image reconstructed in (**g**). The region excluded for quantification is shadowed. Scale bars in (**g–j**) are 20 μm. (**k**) Mean fluorescence for Hh in the simple projection case (Projected, no-flattening, black), and in the flattened eye disc, before and after contour length correction (blue and cyan, respectively) as a function of tissue/contour length. (**l**) Normalized fluorescence of Hh for the whole portion of tissue shown in (**g**) as a function of the distance from the fold center. The same three cases of panel (**k**) are shown. For all the cases, the curves were aligned to the center of the fold before averaging.

Standard analysis methods to quantify a protein concentration in images of immunostained tissue rely on arbitrary choices of the image plane to be quantified or on projections to reduce dimensionality. However, as described above, both of these methods would give rise to artifacts when applied to a curved epithelial tissue. To overcome these limitations, we developed an analysis pipeline to quantify the concentration of a protein in an epithelium presenting a curved shape. The input is a three-dimensional image stack of the tissue, immunostained for the protein of interest (here Hedgehog) (Fig. 2a,g). To apply the pipeline, the following conditions are required: the tissue should be formed by a single cell layer and the apical side of the cells should be identifiable via a marker, such as E-cadherin in *Drosophila* epithelia. For each x–z plane, the profile of the apical edge is obtained by identifying the z-location of the pixels with the highest intensity. From the obtained profile, we extracted relevant information regarding the shape of the tissue, such as the location and width of a fold (Fig. 2b,h), and the length of the apical profile, from now on named contour length (Fig. 2c). To quantify the pixel intensity of the immunostained protein only inside the tissue, first, the apical profile was computationally flattened (Fig. 2d,i, Supplementary Fig. S1). Second, a region of interest was identified as a rectangle of fixed height with the top side overlapping the flattened apical edge (Fig. 2d,j). The average pixel intensity for each x-location was computed and expressed as a function of the contour length (Fig. 2e,k, Supplementary Fig. S1). Finally, all the obtained curves, resulting from the analysis of single x–z planes, were aligned to a reference location (here, for instance, the center of the fold) and averaged over y (Fig. 2f,l).

### The slope of the Hedgehog morphogen gradient is comparable between *Drosophila* eye and wing discs

**Figure 3:**
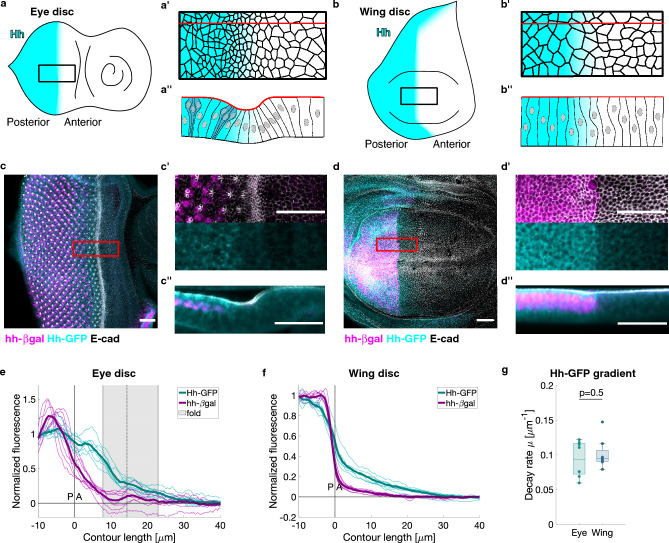
The Hedgehog gradient is similar in the eye and the wing disc. (**a–b′′**) Schematic representations of eye (**a–a′′**) and wing (**b–b′′**) discs illustrating the Hh gradient. (**a′, b′**) show top views and (**a′′, b′′**) show side views of a region of interest at the PA boundary in the discs in which cells outlines are indicated. (**c–d′′**) Representative examples of the Hh distribution (Hh-GFP) and *hh* expression (hh-β-gal) in eye and wing discs. Discs were also immunostained for E-cadherin (grey) to identify the apical edge. Scale bars are 20 μm. (**c, d**) z-projection (maximum for E-cadherin and β-gal, mean for Hh-GFP). (**c′, d′**) z-projection (as before) of a region of interest at the PA boundary in the disc. (**c′′, d′′**) y-projection (mean) of a region of interest (20 μm). (**e–f**) Normalized Hh expression profile (hh-β-gal) and gradient (Hh-GFP) as a function of contour length relative to the position of the PA boundary for N = 8 eye (**e**) and N = 8 wing (**f**) discs. Pairs of wing and eye discs were extracted from the same larvae. Single curves (dotted) and averages (thick) are shown. (**g**) Decay rates extracted by single exponential fitting of the Hh-GFP curves in the anterior compartment. *P*-value obtained from a two-sample t-test.

To test whether specific tissue morphologies, such as epithelial folds, influence morphogen distribution, we applied the analysis pipeline to measure the Hedgehog gradient in the *Drosophila* eye and wing discs (Fig. 3a,b). The Hedgehog distribution was visualized in the discs by using a GFP-tagged version of Hedgehog (Hh-GFP)^46^. The Hh-GFP gradient was comparable with the gradient of the endogenous Hedgehog protein (Supplementary Fig. S2). Moreover, the Hedgehog gradient, as identified in our pipeline in the apico-lateral region of the wing disc, resembled the predominantly baso-lateral gradient previously observed by extracellular staining of Hedgehog^46,57^ (Supplementary Fig. S3). The region of *hh* gene expression was identified using a *hh-lacZ* reporter, which expresses β-galactosidase under the *hh* promoter^49^. Apico-lateral cell edges were identified by immunostaining for E-cadherin, a component of adherens junctions. Late third-instar larval wing and eye discs were fixed and immunostained for E-cadherin, β-galactosidase and Hh-GFP (Fig. 3c,d). We acquired three-dimensional image stacks and analyzed them according to our pipeline. We used the β-gal signal to identify the posterior-anterior (PA) compartment boundary, which we used as a reference location for alignment (Fig. 3e,f). Our pipeline resulted in a more consistent quantification of the Hh-GFP gradient in the eye disc compared to other analysis approaches (Supplementary Fig. S4). Despite a different level of *hh* expression (Supplementary Fig. S5), the Hh-GFP gradient in the eye disc had a comparable shape to the one in the wing disc (Fig. 3e,f), as confirmed by the lack of a significant difference in the decay rate obtained by exponential fitting (Fig. 3g, Supplementary Fig. S5). However, the *hh* expression profile in the eye disc presented a slower decay than in the wing disc (Fig. 3e–f, Supplementary Fig. S5). Thus, the Hedgehog morphogen gradient is comparable in eye and wing discs, despite different expression profiles and different tissue morphologies.

### Interfering with the fold formation in the eye disc leads to a shift in the Hedgehog distribution

**Figure 4:**
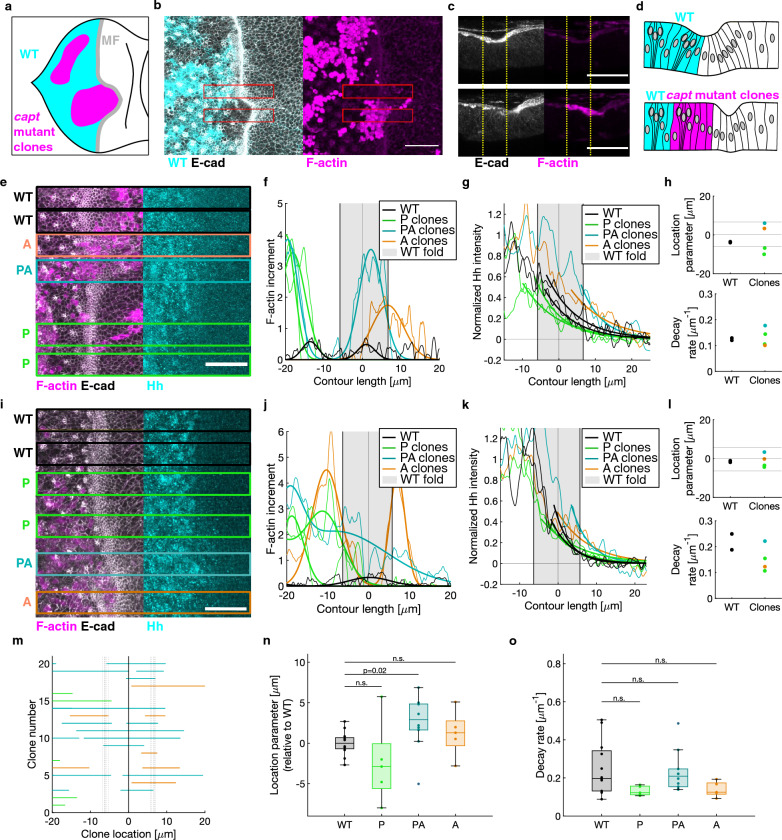
The presence of *capt* mutant clones in the morphogenetic furrow (MF) area leads to a shift in the Hedgehog distribution compared to wild type (WT). (**a–b**) Schematic representation (**a**) and maximum z-projection (**b**) of images of an eye disc stained for E-cadherin (grey) and F-actin (magenta) where clones mutant for *capt* have been induced. Clones are identified by elevated F-actin levels. (**c**) y-projection (mean) of regions of interest (red rectangles in **b**) corresponding to wild-type (top) and *capt* mutant clone at the PA boundary (bottom). Location of the fold in the wild-type region is indicated by the dotted yellow lines. Scale bars are 20 μm. (**d**) Schematic representations of (**c**). (**e–l**) Representative examples of eye discs with *capt* mutant clones and analysis. (**e, i**) z-projections (max). Regions of interest used for analysis are shown (wild-type in black, P clones in green, PA clones in cyan, and A clones in orange). Each region of interest corresponds to a curve or point in the following graphs. (**f, j**) Increment of F-actin relative to wild-type areas of the tissue, expressed as a function of the contour length. Thick lines represent Gaussian fits (single or double) used to identify the clone location. (**g, k**) Normalized Hh fluorescence (thin lines). The thick lines represent the exponential fits. (**h, l**) Parameters resulting from the sigmoidal and exponential fits to the Hh distribution in **g** and **k**. Top: Location parameter, i.e. inflection point from the sigmoidal fit; average location of the start, center and end of the fold in wild-type are shown as black lines. Bottom: Decay rate obtained by exponential fitting. (**m**) Summary of the clones’ categorization. For each analyzed clone (n = 21 regions of interest, N = 6 eye discs) the area covered by the clone is represented with a line. Color code according to the categorization as before. (**n**) Location parameters resulting from the sigmoidal fits on the Hh profiles for all the regions of interest, analogous to (**h**) and (**l**) (top). (**o**) Decay rates resulting from the exponential fits of the Hh profiles for all the regions of interest with clones, analogous to (**h**) and (**l**) (bottom). Statistical significance was tested by a two-sample t-test (two-tailed, n.s. *p* > 0.05).

To test whether the shape of the Hedgehog gradient in the eye disc is dependent on the presence of the morphogenetic furrow we interfered with its formation by inducing clones of cells mutant for *capt*, a gene encoding a negative regulator of actin polymerization^58^. As shown previously^59^, in *capt* mutant clones, F-actin was strongly enriched and, if clones were overlying the morphogenetic furrow area, no fold was present (Fig. 4a–d). However, a fold continuous with the morphogenetic furrow was formed anterior to the clone (Fig. 4a–d).

To reveal whether this local loss of the fold has an impact on the Hedgehog distribution, we immunostained eye discs carrying *capt* mutant clones for the endogenous Hedgehog protein, together with F-actin, to identify the clones, and E-cadherin, to identify the apical edge of the tissue (Fig. 4e,i). The immunostainings were performed two days after induction of *capt* mutant clones. Therefore, by the time the Hedgehog gradient reaches the region of a clone, the clone cells will be unable to form the morphogenetic furrow. We applied our image analysis pipeline to different regions of interest in the eye disc. We categorized the clones according to their location relative to the morphogenetic furrow (Fig. 4f,j,m): Posterior (P) clones were defined as the ones located on the posterior side of the morphogenetic furrow, Posterior-Anterior (PA) clones as the ones covering most of the morphogenetic furrow area, including the center of the morphogenetic furrow, and Anterior (A) clones as the ones touching the morphogenetic furrow area on the anterior side. We compared the Hedgehog distribution in regions of interest corresponding to the different types of clones and wild-type within the same eye disc (Fig. 4). Here, two representative examples of eye discs with *capt* mutant clones in different locations are shown (Fig. 4e–h and Fig. 4i–l, respectively). In both cases, two regions of interest correspond to P clones, one region to a PA clone and one region to an A clone (Fig. 4e–f, i–j). Hedgehog localized more posteriorly or equal to wild-type in presence of P clones, more anteriorly in presence of PA clones and more anteriorly or equal to wild-type in presence of A clones (Fig. 4g,k). To quantify these results, we fitted a sigmoid to the Hedgehog profile (Supplementary Fig. S6) and extracted the location parameter (Supplementary Fig. S6, and Fig. 4h,l, top), which describes the inflection point of the curve. We interpret this location parameter as the boundary between the source and target tissue of Hedgehog. Then, we fitted a single exponential to the Hedgehog profile only in the target tissue (Fig. 4g,k and Supplementary Fig. S6) to obtain the decay rate of the gradient (Fig. 4h,l, bottom). The same type of analysis was applied to N = 6 eye discs which presented *capt* clones in different locations (Fig. 4m, Supplementary Fig. S6). We found that the location parameter was significantly higher, i.e. more anterior, in the case of PA clones (Fig. 4n). However, the decay rate remained unchanged, when compared to wild-type, for all types of clones (Fig. 4o). Thus, interfering with F-actin depolymerization in the area corresponding to the morphogenetic furrow leads to a shift in the Hedgehog distribution to the anterior, rather than a change in the decay rate of the Hedgehog gradient.

### Inducing an ectopic fold at the PA boundary of the wing disc does not affect the Hedgehog gradient

**Figure 5:**
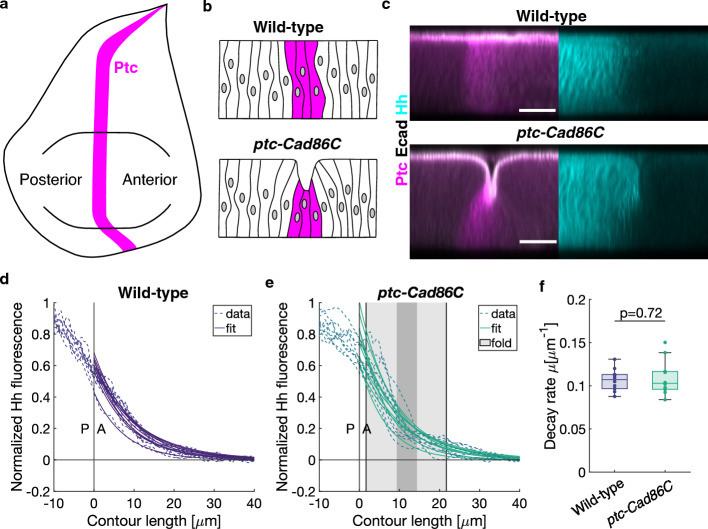
Inducing an ectopic fold at the PA boundary of the wing disc does not affect the slope of the Hedgehog gradient. (**a**) Schematic representation of a wing disc showing the Ptc-expression domain (identifying the PA boundary). (**b**) Schematics of the side view of the wing disc for wild-type (top) and *ptc-Cad86C* (bottom). Cell outlines and Ptc domain are shown. (**c**) y-projection (mean) of regions of interest in wild-type and *ptc-Cad86C* wing discs immunostained for Ptc and E-cadherin (left) and Hh (right). Scale bars are 20 μm. (**d–e**) Normalized Hh fluorescence relative to the contour length (PA boundary set as 0) for N = 10 wild-type and N = 10 *ptc-Cad86C* wing discs. Curves and single exponential fits are shown. Light grey box indicates the range of contour length corresponding to the fold (average). Darker grey box indicates the range of the fold center. (**f**) Decay rate extracted by the fits in panels (**d**) and (**e**). *P*-value obtained from a two-sample t-test.

To test whether the Hedgehog gradient can be affected by the presence of a fold in a different system, we ectopically induced fold formation at the PA boundary of the wing disc (Fig. 5a). We overexpressed Cad86C, a member of the Cadherin family, which can induce fold formation in the wing disc^60^, under the control of the *ptc* promoter (*ptc-Gal4/UAS-Cad86C*), which is active in a stripe of anterior cells along the PA boundary (Fig. 5b). We immunostained *ptc-Cad86C* and wild-type wing discs for E-cadherin and Hedgehog, and for Ptc to identify the PA boundary (Fig. 5c). *ptc-Cad86C*, but not wild-type wing discs, displayed a fold at the PA boundary (Fig. 5b–c). The formation of the fold depends on *ptc-Gal4* expression, which begins in first instar wing discs^61^. Therefore, we dissected third instar wing disc to measure the Hedgehog gradient, assuming that a novel steady state of the Hedgehog distribution in the folded tissue had been reached. We then applied our analysis pipeline to images of *ptc-Cad86C* and wild-type wing discs, using the Ptc expression as a reference for alignment. By fitting a single exponential function to the Hedgehog gradient in the anterior compartment we extracted the decay rate (Fig. 5d–f and Supplementary Fig. S7). No significant difference in the decay rate of the Hedgehog gradient was found between the folded wing discs and control. By contrast, depletion of the Hedgehog receptor Patched (Ptc) in control wing discs resulted in an extended range of the Hedgehog gradient (Supplementary Fig. S8), as previously shown^62^. Therefore, the presence of a fold at the PA boundary does not affect the Hedgehog gradient in the wing disc.

## Discussion

Patterning through morphogen gradients provides cells with positional information during animal development. How positional information is maintained during morphogenetic events, and how these events might contribute to the patterning process remain open questions in the field^14,31^. In this work, we provide an analysis pipeline that enables quantifying the distribution of a morphogen inside curved epithelia. We applied our analysis pipeline to the Hedgehog gradient in the *Drosophila* wing and eye discs, in which the Hedgehog protein disperses through a flat or folded epithelium, respectively. Moreover, we perturbed the shape of the discs by genetic modifications, suppressing fold formation in the eye disc and generating an ectopic fold in the wing disc. We found that the slope of the Hedgehog gradient is not affected by tissue morphology. However, changes in the actomyosin cytoskeleton, which suppress fold formation in the eye disc, resulted in ectopic Hedgehog production. Thus, our work provides a pipeline to quantify protein gradients in curved epithelia and shows that the Hedgehog gradient is robust to variations in tissue morphology.

By labeling simultaneously the cells which express the *hh* gene and the Hedgehog protein itself, we were able to visualize both the Hedgehog protein distribution and its expression profile and compare them between wing and eye discs. We found that while in the wing disc *hh* expression sharply declines, in the eye disc this transition is gradual (Supplementary Fig. S5). We attribute this difference to the fact that in the eye disc the posterior compartment, i.e. the source of Hedgehog, expands in time^50^. Moreover, the expression level of *hh* in the eye disc is lower compared to the wing disc (Supplementary Fig. S5). Taken together, the sources of Hedgehog in the eye and wing disc have different characteristics. On the other hand, we found that the decay rate of the Hedgehog gradient is comparable between the wing and the eye disc ($\sim 0.1\, \upmu {\text{m}}^{ - 1}$), and that it is comparable with previous measurements in *Drosophila* eye discs^63,64^. Thus, the Hedgehog morphogen gradient forms robustly in different *Drosophila* imaginal discs, even when expressed at different levels and in presence of different tissue shapes.

To further test a possible influence of tissue curvature on the Hedgehog gradient, we genetically manipulated the shape of cells to inhibit the formation of the morphogenetic furrow in the eye disc and to induce ectopic folds in the wing disc. Aude et al. previously showed that clones mutant for *capt*, which increase F-actin polymerization, affect cell shape in the morphogenetic furrow and lead to premature photoreceptor differentiation^59^. This result was interpreted either that (i) Hedgehog movement is restricted by the morphogenetic furrow or (ii) F-actin induces a signal leading to premature photoreceptor differentiation. However, in that study, the distribution of Hedgehog was not analyzed. We now observe that clones mutant for *capt*, when covering most of the morphogenetic furrow area, result in a more anterior distribution of Hedgehog compared to wild-type (Fig. 4n). However, no significant change in the slope of the Hedgehog gradient was obtained (Fig. 4o). Similarly, the induction of ectopic folds at the PA boundary of the wing disc did not affect the slope of the Hh gradient (Fig. 5f). Taken together, these results indicate that the shape of the Hedgehog gradient is robust towards epithelial folding in *Drosophila* discs. These results do not lend support to the hypothesis that the morphogenetic furrow restricts Hedgehog movement. However, we note that our approaches (*capt* mutants in the eye disc and Cad86C overexpression in the wing disc) mainly affect the tissue shape at the apical side^60,65^. Such deformation could modify the gradient of a morphogen freely diffusing in an enclosed extracellular space by changing the effective diffusion coefficient^66,67^. However, as Hedgehog is binding to heparan sulfate proteoglycans it is unlikely to diffuse freely^44^. Since it remains controversial whether Hedgehog long-range dispersal in *Drosophila* imaginal discs is mainly on the apical or the basolateral side^45–47,68^, we cannot exclude that affecting the curvature of the basal side of the tissue might change the Hedgehog gradient.

Interestingly, the presence of *capt* mutant clones in the morphogenetic furrow area of the eye disc led to a shift in the Hedgehog distribution in the anterior direction (Fig. 4n). We interpret this shift as an expansion of the source, resulting from ectopic Hedgehog production. As the cells that produce Hedgehog are differentiating photoreceptors^69^, this result indicates that an increase in the levels of F-actin contributes to the differentiation process in the eye disc, consistent with previous observations which linked the modification of the actin cytoskeleton with the speed of morphogenetic furrow progression and photoreceptor differentiation^59,70^. Furthermore, it is known that mechanical stress can induce differentiation, with examples ranging from the *Drosophila* embryo to human stem cells^13,71–76^. Taken together, this evidence allows us to speculate on the role of the morphogenetic furrow in the eye disc: the F-actin enrichment of the cells in the furrow^54,55^ might be a cue for cell differentiation^59^. However, we did not notice any premature differentiation when the cells presenting high levels of F-actin were located in the tissue on the anterior side of the morphogenetic furrow, indicating that F-actin enrichment is not sufficient to trigger cell differentiation. Further investigations are required to understand whether and how the cytoskeletal architecture plays a role in photoreceptor differentiation in the eye disc.

In conclusion, our study provides a new analysis method for quantifying protein distribution in curved epithelial tissues. By applying it to the Hedgehog morphogen gradient in *Drosophila* imaginal discs, we show that the slope of the Hedgehog gradient is robust towards changes in tissue curvature.

## Materials and methods

### Fly stock

Unless stated otherwise, flies were fed on standard food and incubated at 25 °C. Fly stocks used in this work were *hh-sfGFP*^46^ (Bloomington Drosophila Stock Center (BDSC) stock ID #86271, carries a genomic fragment containing *hedgehog* tagged with *sfGFP* on a Bacterial Artificial Chromosome, Hh-GFP is functional^46^), *hh-lacZ*^49^*, hh-Gal4* (described in FlyBase http://flybase.org), *UAS-hh-GFP*^57^*, capt[E593], FRT40A* (BDSC #5942)*, ey-FLP; GMR-myr.GFP, FRT40A* (BDSC #7111)*, ptc-Gal4*^77^*, UAS-Cad86C-HA*^60^*, ap-Gal4*^78^*, tubP-Gal80ts*^79^*, UAS-ds-ci* (Vienna Drosophila Resource Center #51479).

Mosaic clones were generated by Flp-mediated mitotic recombination^80^. The crossing between ;*ap-Gal4, tubP-Gal80ts/BCG;;* and *;; UAS-ds-ci;* was kept at 18 °C and shifted to 29 °C at 3 days before dissection.

For each figure, the genotypes of the larvae were as follows:

- Figure 3, Supplementary Figs. S2, S4, S5: *y w; hh-sfGFP/* + *; hh-lacZ/* + *;*
  *y w; ; ;* (control)
- Figure 4, Supplementary Fig. S6: ey-FLP / w[*] *;* *capt[E593], FRT40​A/GMR-myr.GFP, FRT40A;;*
- Figure 5, Supplementary Fig. S7: *; ptc-Gal4/* + *; UAS-Cad86C-HA/* + *;*
  *;ptc-Gal4/ptc-Gal4 ; ;* (control)
- Supplementary Fig. S3: *; ;* *hh-Gal4/UAS-hh-GFP;*
- Supplementary Fig. S8: *; ap-Gal4, tubP-Gal80ts/* + *; UAS-ds-ci/* + *;*
  *; BCG/+; UAS-ds-ci/+ ;* (control)

### Immunohistochemistry

Late third-instar larvae were dissected in ice-cold phosphate-buffered saline (PBS) solution and fixed for 20 min at room temperature in PBS with 3.7% formaldehyde and 0.2% Triton X-100. The carcasses were washed in PBT (PBS, 0.5% bovine serum albumin, 0.2% Triton X-100) and incubated with primary antibodies overnight at 4 °C. Subsequently, they were washed in PBT and incubated with secondary antibodies for 1 h at room temperature. The carcasses were washed again in PBT and the imaginal discs were dissected and mounted in 50% glycerol, 0.1 M sodium carbonate pH 9, and PPDA (p-phenylene diamine) together with the brain and the mouth hook, which were used as spacers to avoid compression of the tissues. The extracellular staining for Hh-GFP (Supplementary Fig. S3) was based on previously reported extracellular staining protocols^57,81,82^. Late third-instar larvae were dissected in ice-cold Grace’s medium (Sigma-Aldrich, G9771, supplemented with 5% fetal bovine serum, 1% penicillin–streptomycin and 20 nM Ecdysone) and incubated for 1 h at 4 °C in Grace’s medium with the primary antibody (rabbit anti-GFP antibody 1:200). The carcasses were then washed with medium and fixed with 4% paraformaldehyde (30 min at 4 °C and 30 min at room temperature). Subsequent washes, secondary antibody staining and mounting were performed as in the standard staining protocol.

The samples were imaged with a ZEISS Laser Scan 880 (Fig. 3) or ZEISS Laser Scan 980 (Figs. 2, 4, 5) confocal microscope. For all experiments, a 40 × objective was used and images were acquired in z-stacks with pixel size $0.21 \,\upmu {\text{m}}$ × $0.21\,\upmu {\text{m}}$ × $0.45 \,\upmu {\text{m}}$ (x, y, z). For experiments in Figs. 3 and 4, the same image was acquired 4 times and a simple average was performed to reduce noise.

Primary antibodies used in this study were mouse anti-Ptc (1:40, Developmental Studies Hybridoma Bank (DSHB)), mouse anti-*β*-gal (1:200, Promega), rabbit anti-GFP (1:500, Living colors) to detect Hh-GFP, rabbit anti-Hh^83^ (1:500) and rat anti–DE-Cad^84^ (DCAD2, 1:100, DSHB). Secondary antibodies were Alexa-488-, Alexa-594-, Cy5-, and Cy3- conjugated anti-rabbit, anti-mouse, or anti-rat IgG (1:200, Invitrogen). Phalloidin-Alexa-488 (1:100, Molecular Probes, Invitrogen) was used for F-actin staining. DAPI (4′,6-diamidino-2-phenylindole, 1:10,000, Molecular Probes) was used as nuclear counterstain.

### Analysis pipeline

To extract the distribution profile of the protein of interest, firstly, we identified regions of interest at the boundary between posterior and anterior compartments using Fiji^85^. For each type of experiment, a different criterion in selecting the regions of interest was applied (see later). We used the function *Reslice* of Fiji to obtain 3-dimensional images which we further analyzed with a custom-made code in MATLAB (www.mathworks.com).

For each x–z plane, we identified the apical side of the tissue from the signal in the E-cadherin channel. To do so, for each column we found the 5 pixels with the highest intensity and extracted their average height, i.e. z-location. In this way, we were able to express the apical profile as a function of the x-axis of the image: ${\text{a}}({\text{x}}) = \frac{1}{5}\sum\nolimits_{{{\text{i}} = 1}}^{5} {{\text{Z}}_{{\text{i}}} ({\text{x}})}$ where ${\text{Z}}_{{\text{i}}} {\text{(x)}}$ is the height of the i-est pixel with the highest intensity. Note that ${\text{x}}$ is a discrete variable and corresponds to the pixel number of the image ${\text{x}} \in [1, {\text{L}}_{{\text{x}}} ]$ where ${\text{L}}_{{\text{x}}}$ is the size of the image’s x-axis in pixel. For tissues presenting a fold, we identified its position by applying the peak finder function in MATLAB to ${\text{a}}({\text{x}})$ and selecting the peak with the largest width in the vicinity ($\pm \,50\,{\text{ pixels}}$) of the fold location in the previous x–z plane (starting point was provided manually). The location of the peak was named the center of the fold (${\text{x}}_{{\text{F}}}$) and the start (${\text{x}}_{{\text{S}}}$) and the end of the fold (${\text{x}}_{{\text{E}}}$) corresponded to half of the peak height. In all cases, we deformed the image to artificially flatten the tissue. To do so, we shifted each column along the z-axis to align the apical edge to the maximum, taken as a reference: ${\text{a}}^{\prime } ({\text{x}}) = {\text{a}}({\text{x}}) + \Delta {\text{a}}$ where $\Delta {\text{a}} = \max \left( {{\text{a}}\left( {\text{x}} \right)} \right){ - }{\text{a}}({\text{x}})$. We verified that the projection of the resulting image was the same as the one of the original (Supplementary Fig. S1). Then we cut a region of interest of standard height (see later) and averaged the signal of the protein of interest in each column to obtain the protein distribution ${\text{p}}({\text{x}})$. We extracted the contour length by computing the Euclidean distance over the apical profile (Supplementary Fig. S1): ${\text{d}}({\text{x}}) = \sqrt {\Delta {\text{x}}^{2} + (({\text{a}}({\text{x}}){ - }{\text{a}}({\text{x}}{ - }1))\Delta {\text{z}})^{2} }$ where $\Delta {\text{x}} = 0.21\,{\upmu }{\text{m}}$ and $\Delta {\text{z}} = 0.45\,\upmu {\text{m}}$ and expressed the protein distribution according to the contour length, setting ${\text{p}}\left( {\text{l}} \right) = {\text{p}}({\text{x}})$ with ${\text{l}}({\text{x}}) = {\text{l}}({\text{x}}{ - }1) + {\text{d}}({\text{x}})$. Given that the PA boundary is not straight, for the experiments in Figs. 3 and 5 we used an independent measure to align the intensity profiles obtained by each x–z plane (see later). The reference for alignment was also set as the 0 of the x-axes ${\text{l}}^{\prime } = {\text{l}} - {\text{l}}_{{{\text{ref}}}}$. Finally, we averaged the curves over the whole region of interest: ${\text{P}}\left( {{\text{l}}^{\prime } } \right) = \frac{1}{{{\text{L}}_{{\text{y}}} }}\sum\nolimits_{{{\text{i}} = 1}}^{{{\text{L}}_{{\text{y}}} }} {{\text{p}}_{{\text{i}}} ({\text{l}}^{\prime } )}$ where ${\text{L}}_{{\text{y}}}$ is the size in pixel of the image y-axis.

### Quantification of immunostainings in wild-type discs (Fig. 3, Supplementary Figs. S2, S4 and S5)

In order to quantify the distribution of the Hh protein in the eye and wing discs we applied the above-described analysis pipeline to confocal images of fixed discs extracted from larvae expressing *hh-GFP* and *hh-LacZ* and stained for GFP, β-gal and E-cadherin. We cut regions of interest of 400 × 300 pixels around the PA boundary, identified through the *β*-gal staining signal. The height of the region of interest after flattening of the apical edge of the tissue was set to 15 μm (z). In Supplementary Fig. S4 results in absence of tissue flattening are shown. To account for the curvature of the PA compartment boundary we used the β-gal signal as a reference and aligned the curves extracted by single x–z planes before averaging over y. This was done by fitting a sigmoid function (${\text{f(l)}} = \frac{{\text{A}}}{{1 + {\text{e}}^{{\upmu ({\text{l}}{ - }{\text{l}}_{0} )}} }}$) to the averaged *β*-gal fluorescence intensity and taking the location parameter as the reference for alignment. We applied the same analysis pipeline to discs extracted from *yw* larvae, which we used as negative control in each experiment. We observed that the fluorescence intensity for both the GFP (Hh-GFP) and Cy3 (hh-*β*-gal) channels were not showing a flat profile as expected, probably due to the fact that in the fold region the tissue is farther from the objective. To correct for this effect, we quantified the average fluorescence intensity in control discs (*yw* larvae) as a function of the contour length (${\text{p}}_{{{\text{WT}}}} ({\text{l}})$). Then, we used the average fold location as a reference to align the curves (${\text{l}}^{*} ({\text{x}}) = {\text{l}}({\text{x}}) - {\text{l}}_{{\text{F}}}$) and the control intensity was subtracted to the data ${\text{p}}_{{{\text{corr}}}} ({\text{l}}^{*} ) = {\text{p}}({\text{l}}^{*} ) - {\text{p}}_{{{\text{yw}}}} ({\text{l}}^{*} )$. The same step was applied also to the curves from the wing discs for consistency. Then, we performed an average as previously described and the curves were expressed relative to the contour length, setting the PA boundary location as 0 ${\text{P}}\left( {{\text{l}}^{\prime } } \right) = {\text{P}}({\text{l}}{ - }{\text{l}}_{{{\text{PA}}}} )$. Finally, in order to make the intensity profiles comparable between different experimental replicates, the curves were normalized: ${\text{P}}_{{{\text{norm}}}} {\text{(l}}^{\prime } {)} = \frac{{{\text{P(l}}^{\prime } {)}{ - }{\text{P}}_{{\text{A}}} }}{{\frac{{1}}{{{\text{L}}_{{{\text{source}}}} }}\mathop \sum \nolimits_{{{\text{source}}}} {\text{P}}\left( {{\text{l}}^{\prime } } \right){ - }{\text{P}}_{{\text{A}}} }}$ where ${\text{P}}_{{\text{A}}}$ is the average intensity in the extreme anterior of the region of interest (${\text{l}}^{\prime } > {40}$ μm) and the source defined as the posterior compartment (${\text{l}}^{\prime } < 0$ ). To quantify the difference in the *hh* expression profiles between eye and wing disc, a sigmoid function was fitted to the hh-*β*-gal curves (Supplementary Fig. S5). To extract the decay rate of the Hh gradient a single exponential function (${\text{f}}({\text{l}}^{\prime } ) = {\text{Ae}}^{{ -\upmu {\text{l}}^{\prime } }}$) was fitted to the curve in the anterior compartment (${\text{l}}^{\prime } > 0$) (Supplementary Fig. S5). A two-samples t-student test was performed as statistical analysis. For Supplementary Fig. S2, we applied the same pipeline but we estimated the PA boundary manually from the Hh-GFP or Hh staining.

### Quantification of extracellular immunostainings in wing discs overexpressing Hh-GFP (Supplementary Fig. S3)

In order to quantify the distribution of the Hh-GFP protein in the baso-lateral extracellular space of the wing disc we first obtained confocal images of fixed discs extracted from larvae expressing *hh-Gal4/UAS-hh-GFP* and stained for extracellular GFP and for nuclei with DAPI. We cut regions of interest of 250 × 450 pixels around the PA boundary, identified through the Hh-GFP fluorescence. Then we obtained cross sections with the function *Reslice* of Fiji and excluded the peripodial membrane (identified manually via the DAPI signal) and the area external to the tissue on the basal side (the limit was found through the extracellular GFP signal). We quantified the average GFP signal in the apico-lateral side (first 20 μm) and the average baso-lateral extracellular GFP (last 15 μm) as shown in Supplementary Fig. S3. We aligned the curves to the PA boundary and performed a single exponential fit in the anterior side of the tissue to extract the decay rate.

### Quantification of immunostainings in *capt* mutant clones in the eye disc (Fig. 4, Supplementary Fig. S6)

In order to observe the effect of the presence of a *capt* mutant clone on the Hh distribution, we firstly selected different regions of interest (standard size $250 \times 50\,{\text{ pixels}}$) of the eye disc according to the presence or absence of a clone, identified through the F-actin enrichment.

We selected two wild-type regions in each eye disc, as the ones that showed no F-actin increment in the vicinity of the PA boundary. In these regions, we identified the average fold location as described above. To determine the location of the clones in a standardized manner, we computed the F-actin increment relative to wild-type, defined as $\frac{{{\text{A}}_{{{\text{clone}}}} ({\text{l}}) - {\text{A}}_{{{\text{WT}}}} ({\text{l}})}}{{\sqrt {\Delta {\text{A}}_{{{\text{clone}}}} ({\text{l}})^{2} + \Delta {\text{A}}_{{{\text{WT}}}} ({\text{l}})^{2} } }}$ where ${\text{A}}({\text{l}})$ is the average fluorescence intensity in the F-actin channel and $\Delta {\text{A}}({\text{l}})$ is the standard error over the region of interest. Then, we fitted to it single (${\text{f}}({\text{l}}) = {\text{Ae}}^{{{ - }{\text{B}}({\text{l}}{ - }{\text{l}}_{0} )^{2} }}$) or double (${\text{f}}({\text{l}}) = {\text{A}}_{1} {\text{e}}^{{ - {\text{B}}_{1} ({\text{l}} - {\text{l}}_{0 - 1} )^{2} }} + {\text{A}}_{2} {\text{e}}^{{ - {\text{B}}_{2} ({\text{l}} - {\text{l}}_{0 - 2} )^{2} }}$) Gaussian function, depending on which of these two would give the lowest ${\text{R}}^{{2}}$ (Fig. 4f,j, Supplementary Fig. S6). We categorized the clones according to their location relative to the MF in the wild-type: Posterior (P) clones were defined as the ones located on the posterior side of the fold, Posterior-Anterior (PA) clones as the ones covering most of the MF area, including the center, and Anterior (A) clones as the ones touching the fold on the anterior side (Fig. 4m). We quantified the Hh distribution according to our pipeline, the x-axis was repositioned setting the average fold location in wild-type as 0 (${\text{l}}^{\prime } = {\text{l}} - {\text{l}}_{{\text{F}}}$) and the curves were normalized: ${\text{P}}_{{{\text{norm}}}} {\text{(l}}^{\prime } {) = }\frac{{{\text{P(l}}^{\prime } {)}{ - }{\text{P}}_{{\text{A}}} }}{{\frac{{1}}{{{\text{L}}_{{{\text{source}}}} }}\mathop \sum \nolimits_{{{\text{source}}}} {\text{P}}\left( {{\text{l}}^{\prime } } \right){ - }{\text{P}}_{{\text{A}}} }}{ }$ where $P_{A}$ is the average intensity in the extreme anterior (${\text{l}}^{\prime } > 20\, \upmu {\text{m}}$) and the source is the extreme posterior (${\text{l}}^{\prime } < {-15 }\,\upmu {\text{m}}$) of the region of interest. In absence of an independent measure for the PA boundary localization, we fitted a sigmoid function (${\text{f}}({\text{l}}^{\prime } ) = \frac{1}{{1 + e^{{\upmu ({\text{l}}^{\prime } - {\text{l}}_{0}^{\prime } )}} }}$) to the normalized curves and identified the PA boundary as the location parameter (${\text{l}}_{0}^{\prime }$) (Supplementary Fig. S6). To measure the slope of the Hh gradient, we fitted a simple exponential (${\text{f(l}}^{\prime } {)} = {\text{A}} {\text{e}}^{{{ - }{\mu l}^{\prime } }}$) to the curves only on the anterior side of the tissue (${\text{l}}^{\prime } - {\text{l}}^{\prime }_{0} > 0$) (Supplementary Fig. S6). A two-samples t-student test was performed as statistical analysis.

### Quantification of immunostainings in wing discs overexpressing Cad86C (Fig. 5, Supplementary Fig. S7)

In order to observe the effect of the presence of a fold on the Hh distribution in the wing disc, we quantified the distribution of endogenous Hh in wild-type and *ptc-Cad86C* wing discs and applied our analysis pipeline. We cut regions of interest of $400 \times 300\,{\text{ pixels}}$ around the PA boundary, identified through the Ptc staining signal. The height of the region of interest after image deformation was set to $10 \,\upmu {\text{m}}$ (z). Analogously to before, the location of the PA boundary in each x–z plane was identified by fitting a sigmoid function (${\text{f}}\left( {\text{l}} \right) = \frac{{\text{A}}}{{1 + {\text{e}}^{{{\mu}\left( {{\text{l}} - {\text{l}}_{0} } \right)}} }}$) to the Ptc distribution and the obtained location parameter (${\text{l}}_{0}$) was used as a reference for alignment. The Hh distribution was then normalized: ${\text{P}}_{{{\text{norm}}}} {\text{(l}}^{\prime } {) = }\frac{{{\text{P(l}}^{\prime } {)}{ - }{\text{P}}_{{\text{A}}} }}{{\frac{{1}}{{{\text{L}}_{{{\text{source}}}} }}\mathop \sum \nolimits_{{{\text{source}}}} {\text{P}}\left( {{\text{l}}^{\prime } } \right){ - }{\text{P}}_{{\text{A}}} }}{ }$ where ${\text{P}}_{{\text{A}}}$ and the source are defined as in Fig. 3. As a consequence of the subtraction of intensity in the far anterior (${\text{P}}_{{\text{A}}}$) the normalized curves can acquire negative values in the region of the fold (Supplementary Fig. S7). This is a consequence of lower signal intensity in regions far away from the objective (Supplementary Fig. S7a–c) due to light scattering within the tissue. Therefore, we performed a correction by estimating a correction factor with a linear fit on the average variation in fluorescence in the far anterior of the wing disc (Supplementary Fig S7c). This led to a lower variation in signal intensity over z and a smoother Hh profile in the area of the fold/gradient (Supplementary Fig. S7d–e). Finally, a single exponential fit was performed to extract the decay rate (${\text{f}}({\text{l}}^{\prime } ) = {\text{Ae}}^{{{ - }{\mu\text{l}}^{\prime } }}$). The correction for the distance from the objective procedure reduced the variability in the estimated decay rate (compare Fig. 5f and Supplementary Fig. S7h). A two-samples t-student test was performed as statistical analysis.

### Quantification of immunostainings in wing discs expressing ds-RNA targeting *ci* in the dorsal compartment (Supplementary Fig. S8)

To measure the impact of a local reduction of Ptc on the Hh distribution in the wing disc, we quantified the distribution of endogenous Hh in wild-type (absence of *ap-Gal4* identified via the marker BGC) and *ci* knockdown (*ap-Gal4/UAS-ds-ci*) wing discs and applied our analysis pipeline. Ci is a transcription factor required for high-level Ptc-expression. We cut 2 regions of interest of $150 \times 350\,{\text{ pixels}}$, one ventral and one dorsal, around the PA boundary (identified through the Ptc staining signal). The height of the region of interest after image deformation was set to $20 \,\upmu {\text{m}}$ (z). As before, the location of the PA boundary in each x–z plane was identified by fitting a sigmoid function to the Ptc distribution and the obtained location parameter (${\text{l}}_{0}$) was used as a reference for alignment. The Hh distribution was normalized and a single exponential fit was performed in the anterior side to extract the decay rate. The decay rate obtained from the dorsal compartment was then normalized to the ventral one.

## Supplementary Information

Supplementary Information.

## Data Availability

All data generated or analyzed during this study are included in this published article (and its supplementary information files).
