# Supplementary material for: Hedgehog morphogen gradient is robust towards variations in tissue morphology in Drosophila
Source: Sci Rep. 2023 May 25;13:8454. doi: 10.1038/s41598-023-34632-8 (PMC10213063; doi:10.1038/s41598-023-34632-8)
Supplement: Supplementary file 1 — Supplementary Information. [file 41598_2023_34632_MOESM1_ESM.pdf]

Supplementary material – Hedgehog morphogen gradient is robust towards variations in tissue morphology in *Drosophila*

Giulia Pierini, Christian Dahmann

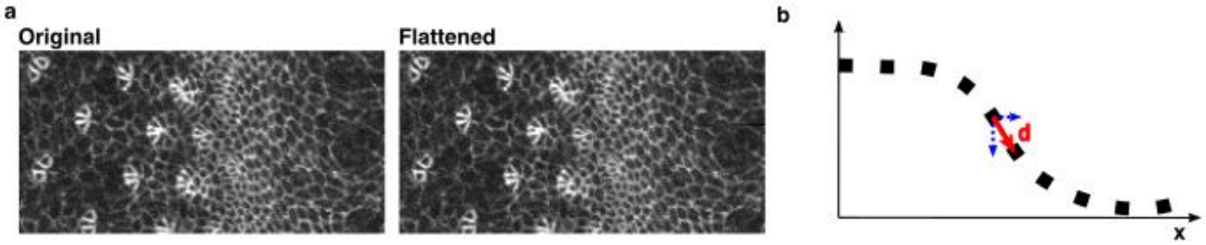

Figure S1: Validation of the flattening procedure and schematics of the contour length computation.

(**a**) z-projection of a section of an eye disc immunostained for E-cadherin before (left) and after (right) flattening. (**b**) Schematics of the Euclidean distance (d, red arrow) over the apical edge. The blue arrows indicate the x and z components of d ($d_x = \Delta x = 0.21$ µm, $d_z = (a(x) - a(x-1))\Delta z$ with $\Delta z = 0.45$ µm).

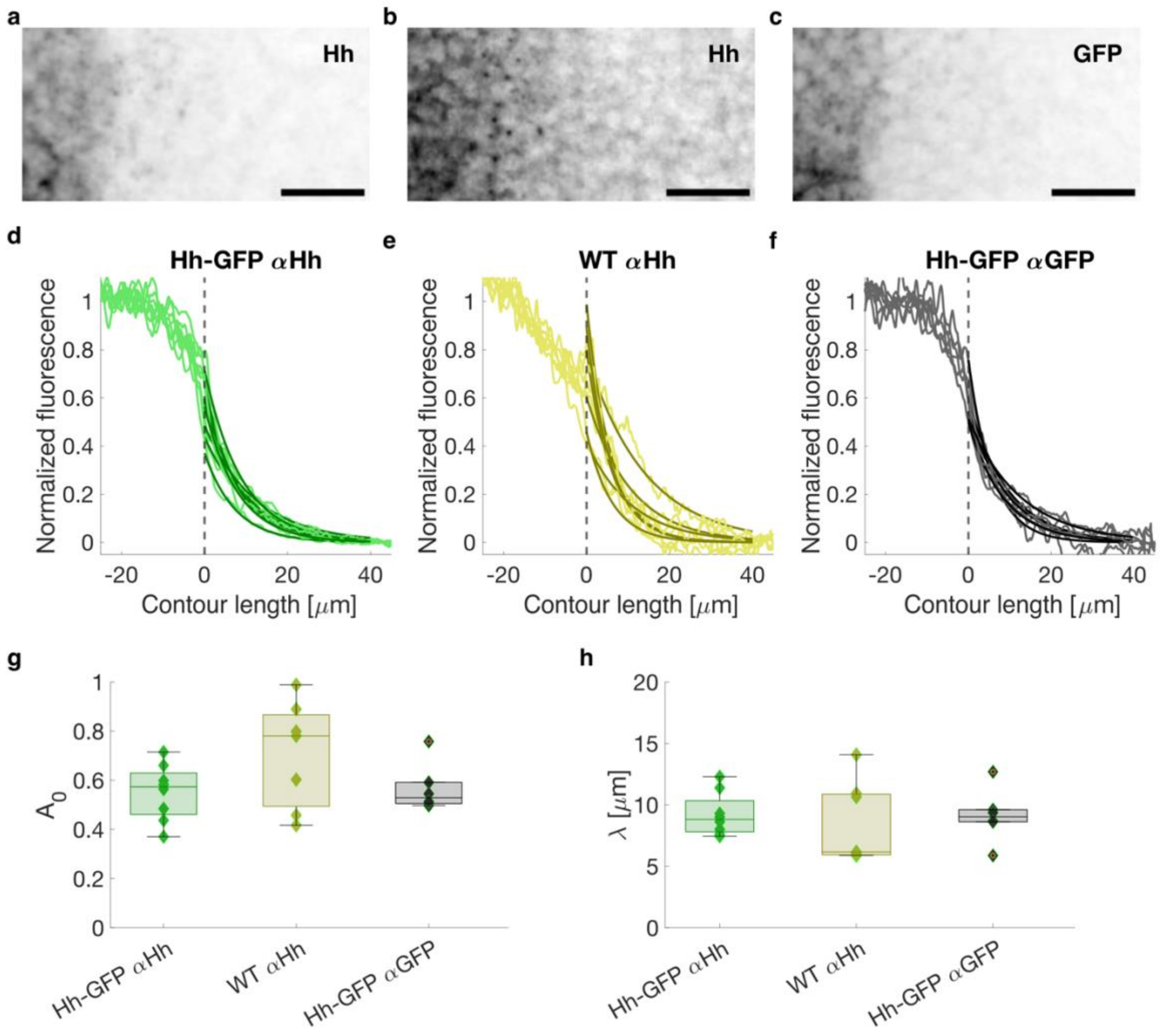

Figure S2: Quantification of the characteristic length of the Hedgehog gradient in the wing disc of wild-type (WT) and Hh-GFP larvae stained for Hh and GFP.

(**a-c**) z-projection (sum) of a region of interest of wing discs expressing Hh-GFP (**a**) or wild type wing discs (**b**) stained for Hh, or wing discs expressing Hh-GFP stained for GFP (**c**). Scale bars are 10 µm. (**d-f**) Normalized fluorescence intensities relative to the contour length resulting from staining for Hh of wing discs expressing Hh-GFP(**d**) or wild type wing discs (**e**), or for staining for GFP of wing discs expressing Hh-GFP (**f**). In each panel, the number of replicates was: N=8 (**d**), N=7 (**e**), N=6 (**f**) wing discs. The x-axis was repositioned setting the PA boundary (estimate) as 0. Darker lines show the result of single exponential fitting in the anterior compartment. (**g-h**) Parameters estimated by single exponential fitting. A_0 is the amplitude of the exponential and λ is the characteristic length of the exponential.

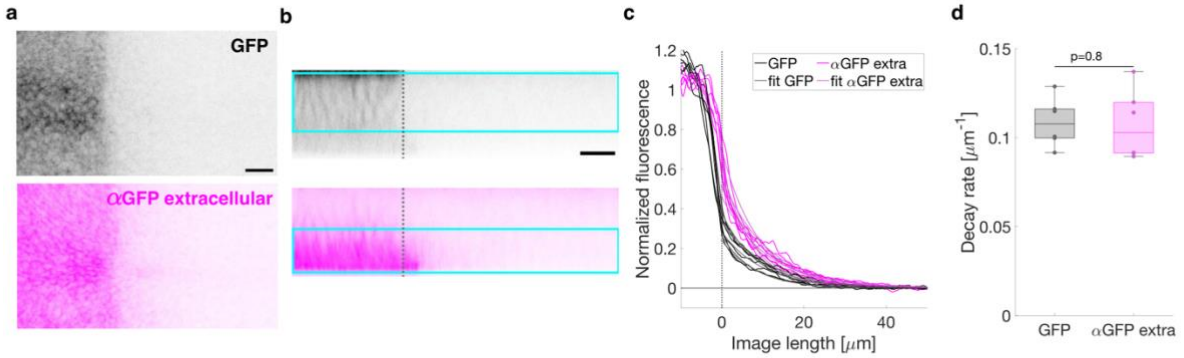

Figure S3: The decay rate of the intracellular Hh-GFP gradient resembles the decay rate of the extracellular Hh-GFP gradient.

(**a**) z-projection (sum) of a region of interest of wing discs expressing Hh-GFP (*hh-Gal4/UAS-hh-GFP*). GFP fluorescence (GFP, grey) and extracellular GFP antibody staining (magenta) are shown. (**b**) y-projection (sum) of a cross-section of the same region of interest shown in the previous panel (**a**). Regions of interest used for quantification are highlighted in cyan (apico-lateral (20 µm height) for GFP and baso-lateral (15 µm height) for extracellular GFP). The dotted lines represent the estimated PA boundary. Scale bars are 10 µm. (**c**) Normalized fluorescence intensities for N=6 wing discs. The x-axis was repositioned setting the PA boundary (estimate) as 0. Lighter lines show the result of single exponential fitting in the anterior compartment. (**d**) Decay rate estimated by single exponential fitting. P-value resulting from a two-sample t-test.

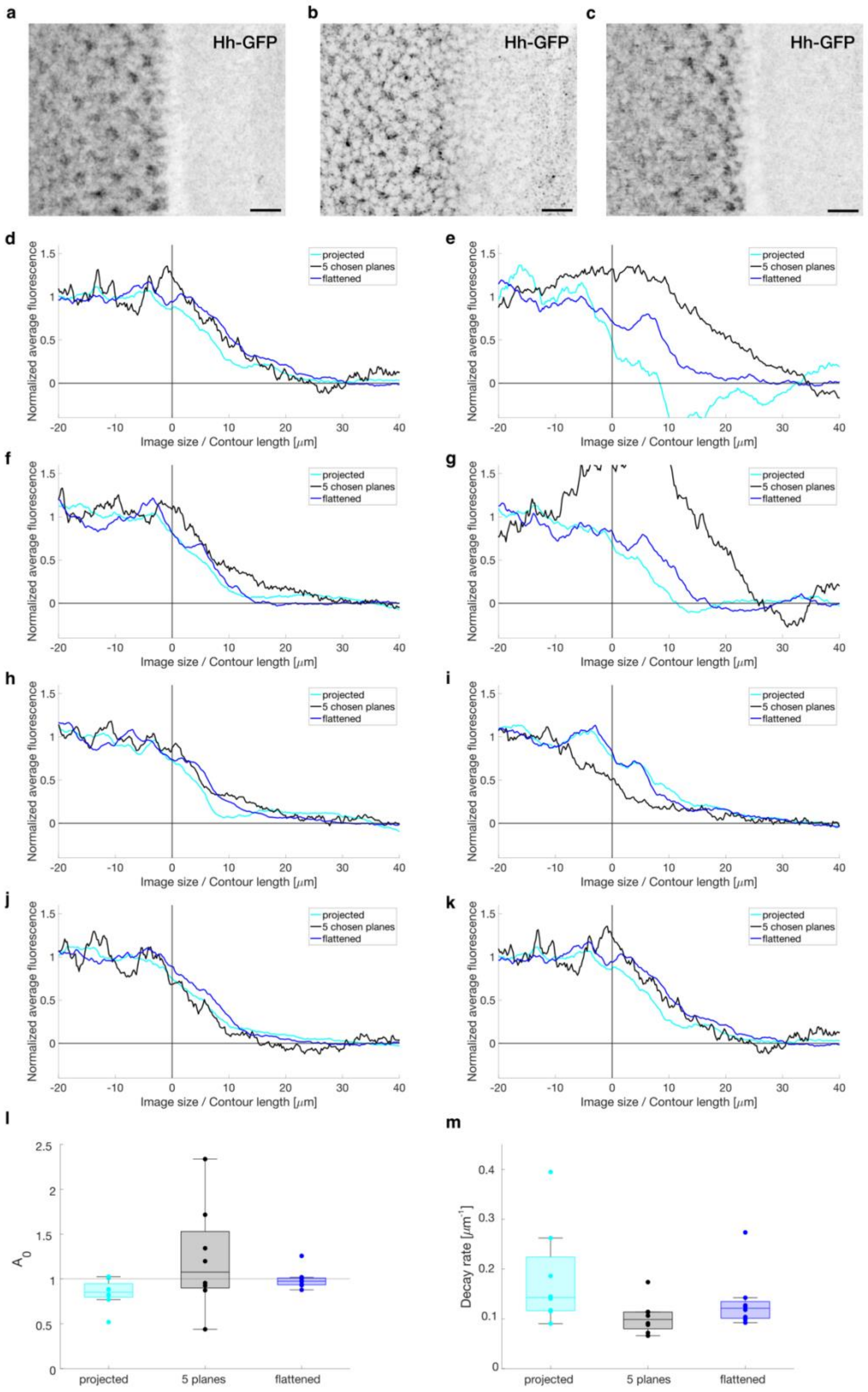

Figure S4: Comparison between different analysis methods: z-projection of the whole image, choice of 5 quantification planes (chosen according to the strongest fluorescence intensity in

the region of the gradient) and our analysis pipeline (flattening and correction for contour length.

(**a-c**) Top views of a region of interest of an eye disc expressing Hh-GFP and stained for GFP. z-projections (sum) (**a**) over the whole image stack, (**b**) over 5 chosen z-planes or (**c**) over z-planes located within 15 µm from the apical side after flattening (**c**). Scale bars are 10 µm. (**d-k**) Normalized fluorescence intensities as a function of contour length relative to the position of the PA boundary for different eye discs obtained from standard z-projection, choice of 5 planes and according to our methodology (flattening and correcting for contour length). The intensities are averaged over the whole region of interest. (**l-m**) Amplitude and decay rate estimated by single exponential fitting of the curves in anterior compartment for N=8 eye discs (same as Fig. 3 main text) are shown.

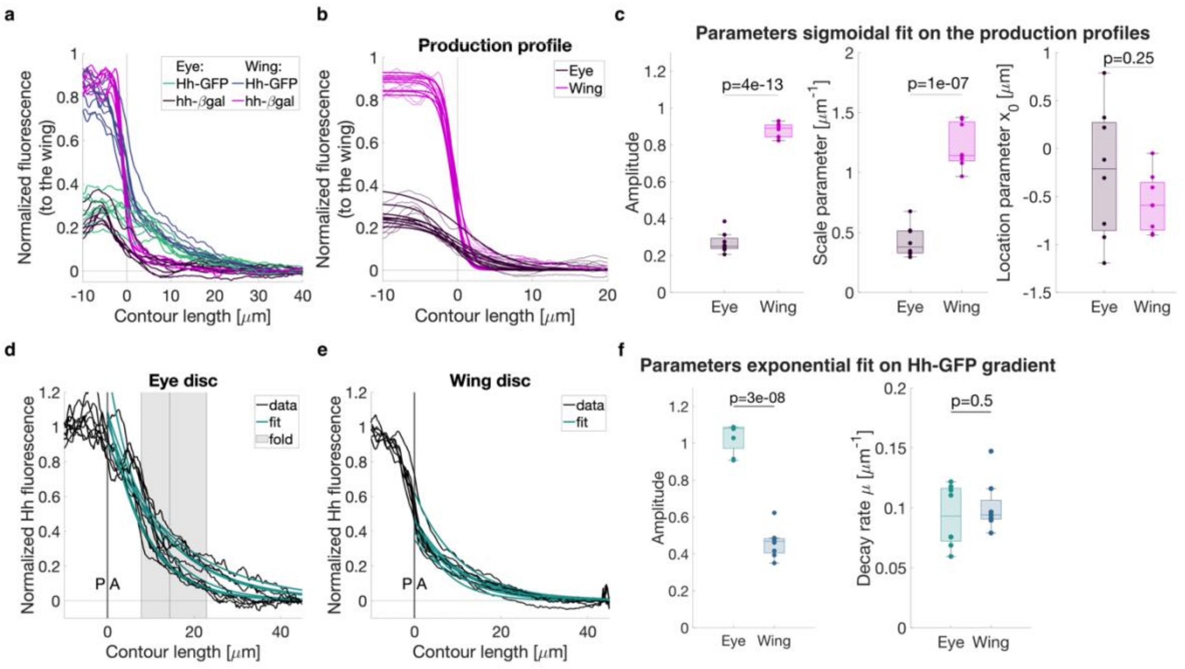

Figure S5: Quantification of the expression level, expression profile and gradient slope for the morphogen Hedgehog in the eye and wing disc.

(**a**) Hh-GFP and hh-βgal fluorescence intensities normalized to the maximum intensity value of the wing and shown as a function of contour length relative to the position of the PA boundary for N=8 eye discs and N=8 wing discs. Pairs of eye and wing discs were extracted from the same larvae. (**b**) Hh expression profile (hh-βgal in **a**): single curves (thin) and sigmoidal fit (thick) are shown. (**c**) Amplitude, scale parameter and location parameter resulting from the sigmoidal fits in panel **b**. (**d-e**) Normalized Hh-GFP fluorescence intensities as a function of contour length for N=8 eye discs (**d**) and N=8 wing discs (**e**). Single curves (black) and exponential fit results (dark cyan) are shown. (**f**) Amplitude and decay rate resulting from the exponential fits shown in panels **d** and **e**. Statistical significance testes by a two-sample t-test.

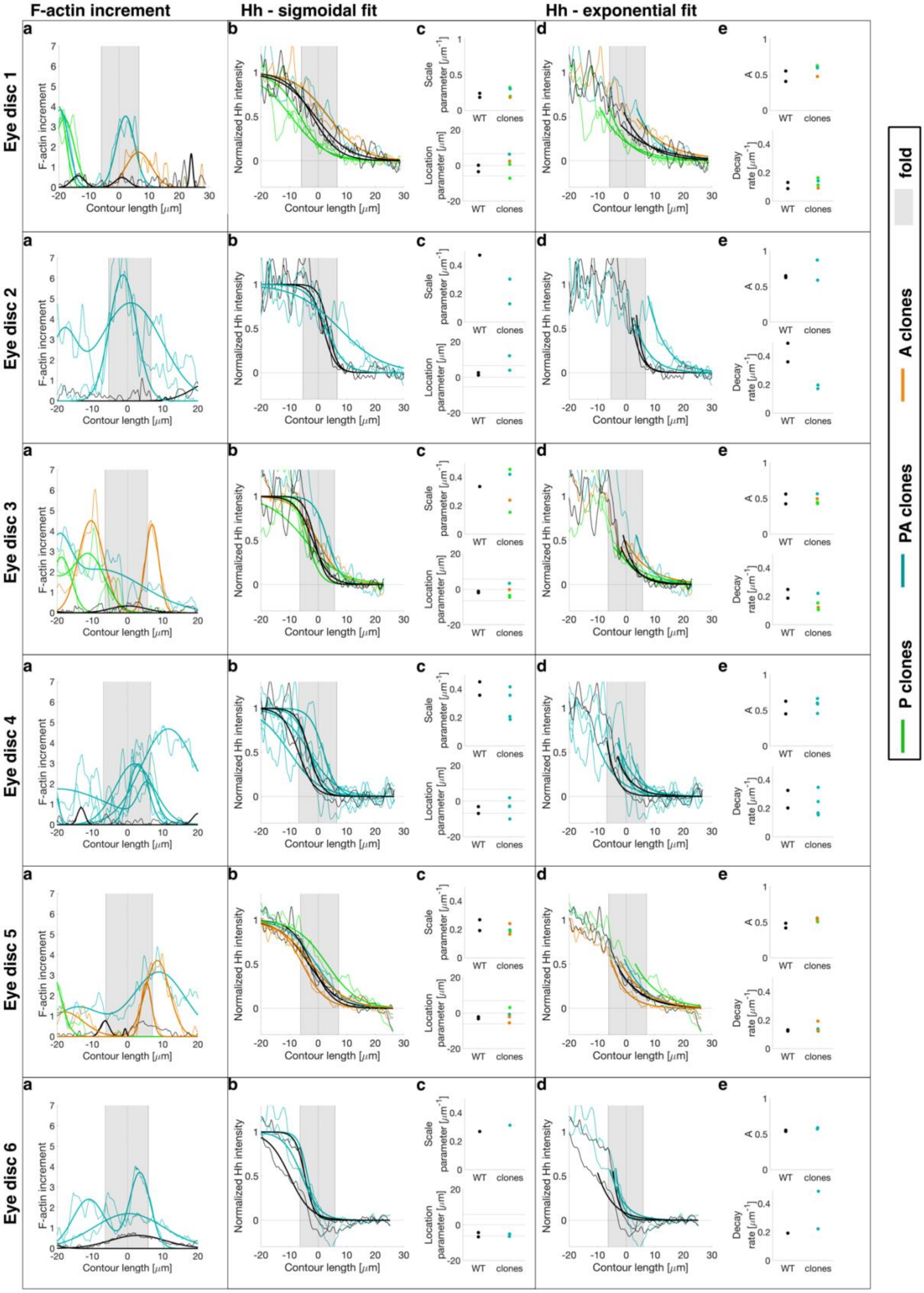

Figure S6: Analysis of N=6 eye discs presenting *capt* mutant clones analogous to (and containing) the examples presented in Fig. 4e-l and resulting in the data shown in Fig. 4m-o.

(**a**) Increment of F-actin as a function of contour length relative to wild-type areas of the tissue, defined as $\frac{A_{clone}-A_{WT}}{\sqrt{\Delta A_{clone}{}^2+\Delta A_{WT}{}^2}}$ where A is the F-actin signal. Thick lines represent the Gaussian fits (single or double) used to identify the clone location relative to the fold and to categorize it as P, PA, or A. (**b**) Normalized Hh fluorescence intensity as a function of contour length (thin lines). Thick lines represent the sigmoidal fits. (**c**) Parameters resulting from the sigmoidal fits in panel **b**: scale parameter (top) and location parameter, i.e. inflection point (bottom). (**d**) Normalized Hh fluorescence as a function of contour length (thin lines). Thick line represents the exponential fit. (**e**) Parameters resulting from the exponential fit in panel **d**: amplitude (top), decay rate (bottom).

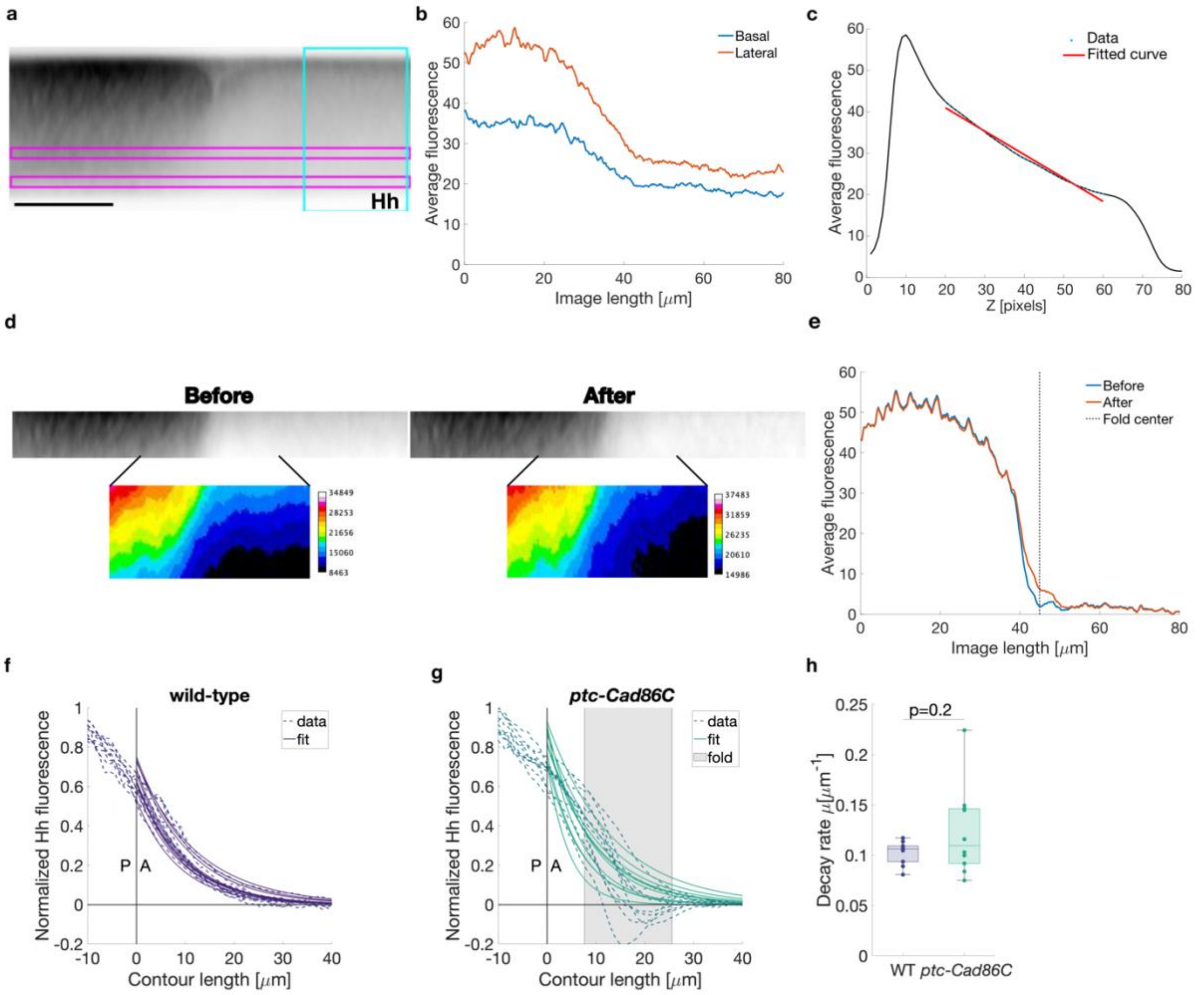

Figure S7: Correction for the distance from the objective in the experiments where we induced a fold in the wing disc (Fig. 5 in the main text).
(**a**) y-projection (sum) of the Hh channel in a region of interest in a *ptc-Cad86C* wing disc. (**b**) Plot of the averaged fluorescence intensity of the two regions of interest shown in magenta in **a** (bottom=basal, top=lateral). (**c**) Variation in background fluorescence relative to the z location in the image. Intensities extracted from the cyan region of interest shown in **a** (far anterior) and averaged over x. To estimate the scaling factor, we performed a linear fit (red line). (**d**) Comparison between regions of interest used for quantification before or after correcting for the distance from the objective. Zoom around the PA boundary is shown and color-coded with the lookup table of 16 colors in Fiji. (**e**) Comparison between the average fluorescence intensity obtained before or after correction for the distance from the objective. The baseline value (far anterior) has been set to 0. (**f-h**) Analogous to Fig. 5d-f in the main text prior to correction for the distance from the objective.

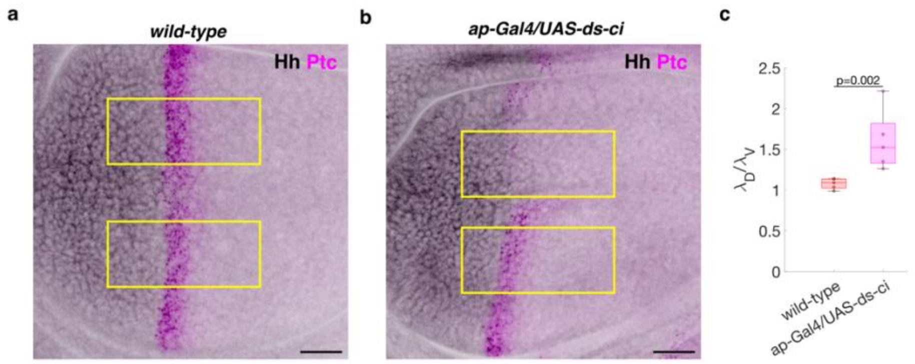

Figure S8: Knockdown of *ci* results in the downregulation of the Hedgehog receptor Ptc and in a significantly extended Hedgehog gradient.
(**a-b**) z-projection (sum) of a region of interest of wild type (**a**) and *ap-Gal4/UAS-ds-ci* (**b**) wing discs stained for Hh and Ptc. The knockdown of *ci* is limited to the dorsal region (to the top), where *ap-Gal4* is active. Regions of interest used for quantification are highlighted in yellow. Scale bars are 20 µm. (**c**) The characteristic length of the dorsal Hedgehog gradient normalized to the ventral one for wild-type (N=5) and *ap-Gal4/UAS-ds-ci* (N=5) wing discs.
